# Potential of helper-dependent Adenoviral vectors in CRISPR-cas9-mediated lung gene therapy

**DOI:** 10.1186/s13578-021-00662-w

**Published:** 2021-07-23

**Authors:** Ranmal Avinash Bandara, Ziyan Rachel Chen, Jim Hu

**Affiliations:** 1 Programmes in Translational Medicine, Research Institute, Hospital for Sick Children, Toronto, ON Canada; 2 Departments of Laboratory Medicine and Pathobiology, University of Toronto, Toronto, ON Canada; 3 University of Toronto, Toronto, ON Canada; 4 The Hospital for Sick Children, Peter Gilgan Centre for Research and Learning, 686 Bay St., Room 09.9715, Toronto, ON M5G 0A4 Canada

**Keywords:** Adenovirus, Gene therapy, Airway gene delivery, Cas9, Cystic fibrosis

## Abstract

Since CRISPR/Cas9 was harnessed to edit DNA, the field of gene therapy has witnessed great advances in gene editing. New avenues were created for the treatment of diseases such as Cystic Fibrosis (CF). CF is caused by mutations in the Cystic Fibrosis Transmembrane Conductance Regulator (*CFTR*) gene. Despite the success of gene editing with the CRISPR/Cas9 in vitro, challenges still exist when using CRISPR/Cas9 in vivo to cure CF lung disease. The delivery of CRISPR/Cas9 into lungs, as well as the difficulty to achieve the efficiency required for clinical efficacy, has brought forth new challenges. Viral and non-viral vectors have been shown to deliver DNA successfully in vivo, but the sustained expression of CFTR was not adequate. Before the introduction of Helper-Dependent Adenoviral vectors (HD-Ad), clinical trials of treating pulmonary genetic diseases with first-generation viral vectors have shown limited efficacy. With the advantages of larger capacity and lower immunogenicity of HD-Ad, together with the versatility of the CRISPR/Cas9 system, delivering CRISPR/Cas9 to the airway with HD-Ad for lung gene therapy shows great potential. In this review, we discuss the status of the application of CRISPR/Cas9 in CF gene therapy, the existing challenges in the field, as well as new hurdles introduced by the presence of CRISPR/Cas9 in the lungs. Through the analysis of these challenges, we present the potential of CRISPR/Cas9-mediated lung gene therapy using HD-Ad vectors with Cystic Fibrosis lung disease as a model of therapy.

## Introduction

Cystic Fibrosis (CF) is an autosomal monogenic recessive genetic disorder that affects over 70,000 individuals in the United States and Europe [1]. Mutations in the Cystic Fibrosis Transmembrane Conductance Regulator (*CFTR*) gene alter its function as a cyclic AMP-dependent chloride anion channel. This protein distributes mainly in epithelial cells of different organ systems, such as respiratory, digestive, and reproductive systems, and mutations in *CFTR* render multiorgan damage in CF patients. In the airway epithelium, the imbalanced ion transport subsequently leads to reduced airway fluid efflux, a thickened mucus layer, and impaired mucociliary clearance, thus creating a mucus-obstructed and inflamed airway. As a result, chronic pulmonary infections make CF life-limiting [2]. Following the discovery of the *CFTR* gene, CF lung disease was considered as a model for lung gene therapy [3].

More than 2000 mutations in the *CFTR* gene have been recorded [4], in which the disease-causing ones are divided into 6 classes. Class I- No CFTR protein is produced due to nonsense or splice mutations. Class II- CFTR protein is not processed and cannot form the right structure or be trafficked to the apical membrane. Class III- Proteins are present at the apical membrane, but the channel remains closed. Class IV- channels are open but ion movement is hindered due to altered internal protein structure. Class V- Not enough proteins are produced to maintain normal function. Class VI- proteins are not stable. Different mutations affect patients to different degrees; individuals with the same genotype have varying disease penetrance as well as responses to treatments. Although traditional treatments of CF include the management of inflammation and mucus overproduction resulted from infection, these treatments can only reduce the symptoms. Current treatments for CF focus on restoring the CFTR protein function [5]. CF modulator drugs that target the CFTR channel function has shown promising results in improving the health for the majority of CF patients [5]. Initially, the first CFTR-modulator, ivacaftor, has been shown to be highly effective for treating patients with a class III mutation, G551D [6] which is present in about 5% of CF patients. Later on, combinations of ivacaftor with other modulators show efficacy in patients with at least one of the class II mutation delta508F, which is present in majority of the CF patients [7]. Nonetheless, treatments for patients with rare CFTR class I mutations still await. In addition, long term effects of the treatments with these new drugs are not totally clear.

Gene therapy, on the other hand, can be a solution for all genotypes and therefore all patients. Gene therapy involves the delivery of a wild type gene into the nucleus to produce normal protein, solving the problem at the origin of the disease. For gene delivery, vectors or vehicles are needed to carry therapeutic genes. These vectors can be divided into two classes, nonviral vectors, such as liposomes and nanoparticles, and viral vectors, such as lentiviral vectors, Adeno-Associated Viral (AAV) vectors and Adenoviral (Ad) vectors.

Liposomes are bilayered, phospholipid nanosized vesicles, and depending on its size can be deemed a nanoparticle [8]. Successful delivery of DNA (a CpG motif-deleted plasmid expressing the human CFTR cDNA) using liposomes to the lungs of CF patients has been accomplished [9], and this is the largest CF gene therapy study using liposomes for CF treatment [9]. There were no adverse effects unique to the treatment observed. However, the study concluded that the efficacy and consistency of the response needed to be improved [9].

Particles described as being less than 100 nm in size are deemed nanoparticles. These include liposomes, iron oxide nanoparticles, polymeric micelles, dendrimers, nanoshells, polymeric nanospheres, nanobins and much more [10]. Currently, over 25 types of nanoparticles have been approved by the Food and Drug Administration (FDA) and the European Medicines Agency (EMA) [10]. However, no nanoparticle carrier has been approved for CF gene therapy [10]. Nanoparticles less than 200 nm in diameter have been shown to penetrate mucus efficiently [11].

Lentiviruses have an RNA genome and can infect both dividing and non-dividing cells. Engineered lentiviral vectors have been shown to transduce into ferret, mouse and sheep airways [12, 13]. These vectors can integrate their cDNA genome into the host chromosomes. However, their safety and efficacy in CF lung gene therapy must be shown.

AAV has been successful in clinical trials for treating hemophilia B, lipoprotein lipase deficiency and night blindness [14, 15]. The major disadvantage of AAV in CF gene therapy is the low DNA carrying capacity of the vector (5 kb) while the *CFTR* minigene is approximately 4.44 kb [14]. Despite this caveat, successful expression of CFTR has been achieved in mice using a more trimmed version of the *CFTR* minigene coupled to a small promoter [15]. Even though CF trials utilizing AAV were unsuccessful due to low expression of CFTR [16], efforts to improve its efficiency in airway gene delivery are continuing.

Ad vectors refer to the first adenoviral vectors used for CF gene therapy. More than 400 gene therapy trials have used human Ad vectors [17, 18]. However, the conventional Ad vectors are not suitable for CF gene therapy because these vectors induce strong host immune responses and the cells transduced with these vectors are eliminated in a couple of weeks [19, 20]. To overcome the problems of these vectors, helper-dependent adenoviral (HD-Ad) vectors have been developed [19]. In these vectors, all viral coding sequences are deleted. Due to the lack of adenoviral genes in the vector genome, these vectors are less toxic and have much larger DNA carrying capacity [19, 21, 22]. Our group has used Adenovirus type-5 based HD-Ad vectors to successfully transduce into primary human cells, mouse airways and pig airways [23]. While HD-Ad vectors based on a different serotype have been used for targeted integration of γ-globin gene in mice for hematopoietic stem cell gene therapy [24], in this review, we will focus on discussing major challenges and possible solutions for using HD-Ad vectors to achieve permanent gene correction in the lungs of CF patients.

### Overcoming challenges of CRISPR/Cas9 delivery

Recent advances in engineering site-specific endonucleases, especially Clustered Regularly Interspaced Short Palindromic Repeats (CRISPR) systems [25] made permanent gene correction possible. CRISPR/Cas9, the most studied gene editing system, increases integration of introduced DNA by creating DSBs (Double Stranded Breaks) and can also edit the existing mutated *CFTR* gene sequences or help regulate CFTR expression (26, 27). When the new tool, CRISPR/Cas9 system, is used in lung gene therapy, new challenges emerge. First, a vector with a large DNA carrying capacity and a high efficiency in airway gene delivery is required to package both the gene expression cassettes of the CRISPR/Cas9 system and the donor DNA with homology arms. Most common gene therapy vectors, such as AAV or Lentiviral vectors, used for in vivo gene delivery do not have the capacity. Our group has shown that the HD-Ad vector can be used to package all components required for achieving highly efficient site-specific gene integration [28, 29]. Although the individual genetic components could also be carried by two vectors, more vectors will reduce the transduction efficiency and induce stronger host immune responses.

As a bacterial protein, Cas9 raises another potential immune challenge. Charlesworth et al. in 2019 detected pre-existing antibodies against both *Staphylococcus aureus* Cas9 and *Streptococcus pyogenes* Cas9 in human serum [30]. The pre-existing immunity to Cas9 could be a potential problem, but it is unlikely a major challenge to gene delivery as pre-existing antibodies to viral vectors do not have a major effect on gene transfer [31]. Additionally, if Cas9 is continuously expressed from gene-modified cells, the host immune system will eliminate these cells, therefore negatively impacting gene therapy efficacy. One solution to this problem is to deliver Cas9 protein or mRNA so that the Cas9 protein presence or expression will be transient. Although this can be achieved in cultured cells, it is difficult to achieve efficient delivery in vivo. Fortunately, HD-Ad vectors have the capacity to deliver both the CRISPR-Cas9 system and donor DNA together and following donor DNA integration, the integrity of the vector genome is compromised, thus leading to the degradation of the residual genome and elimination of Cas9 expression. Thus, this problem can be solved by using HD-Ad vectors [28, 29].

Off-target effects are a concern of all gene-editing tools. The specificity of the CRISPR/Cas9 system relies on single guide RNA (sgRNA) and a Protospacer Adjacent Motif (PAM) next to the target site. However, this concern is often overexaggerated since the rationale for using CRISPR/Cas9 in gene therapy is to take the advantage of its sequence specificity. Positive safety and efficacy results have been obtained in gene therapy treatment of patients with adenosine deaminase immunodeficiency using retroviral vectors which has no integration specificity [32]. This example indicates that the risk of genetic integration in somatic cells is small. Nevertheless, there are many strategies to further enhance the specificity. These strategies include optimizing sgRNA sequence and expression level [33, 34], modifying the Cas9 protein [35] or using a different Cas protein [36]. In addition, the CRISPR system can be modified to perform base editing which does not require a double stranded DNA break. This will be discussed in the next section.

Another major challenge to CRISPR/Cas9-mediated lung gene therapy is the in vivo gene editing (or integration) efficiency because gene delivery in vivo is generally not as efficient as in cultured cells. There are at least four strategies to overcome this problem. First, the most efficient gene therapy vectors can be used to deliver all gene editing components in a single vector. This has been demonstrated by using the HD-Ad vector [28, 29, 37]. Second, small molecules, such as SCR7 [38] may be used to enhance gene integration efficiency. However, when any molecules are used for this purpose, their safety in vivo has to be carefully evaluated. In addition, protein factors that are involved in homology-dependent DNA repair, such as CtIP [39] and inhibitor of 53BP1 [40], have been shown to enhance gene editing. When these factors are used, their expression should be transient to avoid potential problem of affecting cell proliferation. Finally, if a gene expression cassette is used for gene correction, it is possible to integrate this cassette site-specifically in more than one location to achieve higher transgene expression.

### Using CRISPR/Cas9-mediated gene correction to sustain therapeutic gene expression

Expression of functional CFTR proteins in 5 to 15 percent of airway epithelial cells in CF patients is believed to be enough to mimic wild type levels of chloride secretion in vitro [41–43], thus resolving the disease. Although this makes lung gene therapy as an attractive treatment option, it is difficult to achieve sustained therapeutic gene expression in the lung due to the turnover of airway epithelial cells. To overcome this problem, permanent and site-specific gene correction has to be achieved in airway epithelial stem cells. Fortunately, the Hogan group has discovered that some airway basal cells show stem cell properties and can differentiate into all other epithelial types [44, 45]; unlike murine lungs where there are no basal cells, human airway contains a large portion of basal cells. We now know that airway basal cells are heterogeneous, containing both airway stem cells and progenitor cells. Basal cells are located near the basement membrane and are seemingly difficult to transduce. For the first time, our group has demonstrated successful delivery of HD-Ad vectors to pig airway basal cells in vivo and human basal cells from CF patients in air–liquid cultures [23]albeit more research in this area is needed [23].

**Fig. 1:**
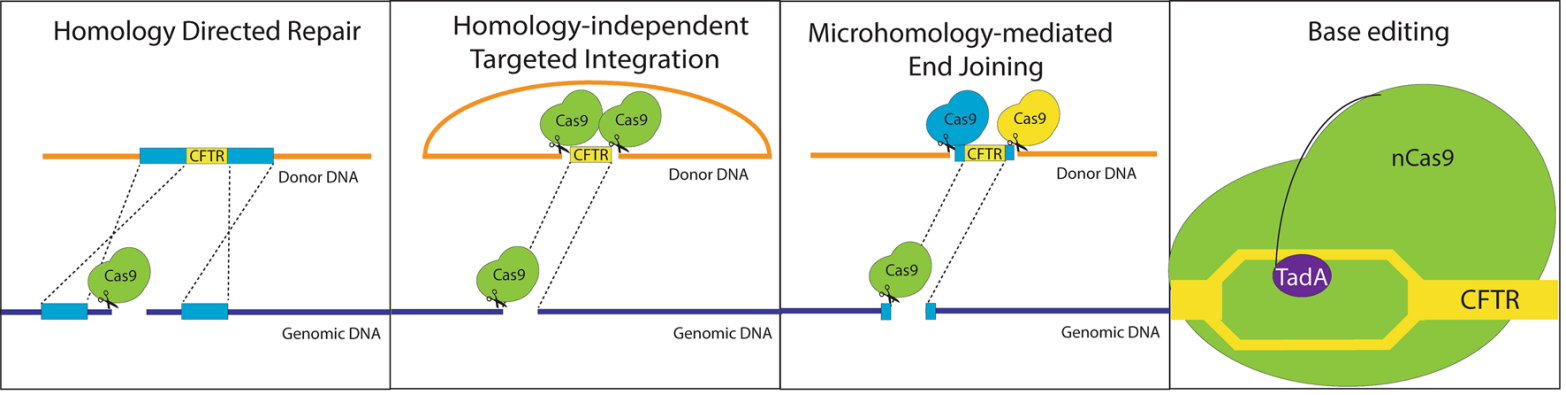
Schematic presentation of four strategies of Cas9-mediated permanent gene correction. Mutant alleles of the CFTR gene can be corrected by integration of a copy of the wild-type gene through homology directed repair or homology independent targeted integration and microhomology-mediated end joining as well as base editing

The CRISPR/Cas9-mediated gene correction is envisioned to play an important role in future lung gene therapy. The following are potential ways that the CRISPR/Cas9 system may be used in gene correction (Fig. 1).

#### Site-specific gene integration

Site-specific insertion of a functional copy of the *CFTR* minigene (cDNA in an expression cassette) in either the *CFTR* locus or a genomic safe harbor, such as AAVS1, would allow long term expression of *CFTR* as it is then replicated and passed to daughter cells. Insertion of DNA is greatly increased when the site of the DNA targeted for integration in the genome is cleaved. In eukaryotes, these cleavage events such as DSBs can be resolved by homologous or non- homologous recombination [46]. Since CRISPR/Cas9 creates DSBs, DNA sequences can be inserted at specific sites using either Homologous Recombination (HR) or Non-Homologous End Joining (NHEJ) [26, 47]. Thus, a *CFTR* gene copy could be integrated into the DNA after CRISPR-mediated DSBs using multiple mechanisms that take advantage of HR or NHEJ [26].

##### Homology directed repair (HDR)

This type of repair results in no mutations occurring at the CRISPR/Cas9 cut site as the DSBs are repaired using a homologous template [48]. CRISPR/Cas9 creates DSBs at specific locations allowing site-specific integration of a functional gene with homology arms complimentary to the target site, and this technique has shown to give successful expression of CFTR in pig cells [9]. Using HDR, up to 10 percent of the cells have been shown to have successful integration of *CFTR* [29]. Furthermore, CRISPR/Cas9-mediated HDR can be further enhanced by factors that could increase HDR over NHEJ [48]. A drawback of this pathway is that non-dividing cells tend not to use HDR and HDR is only active during the late S and G2 stages [49, 50]. Stem cells are a prime target for gene therapy as the correction of a stem cell will result in correction of the mutation to all its daughter cells. However, HDR cannot occur at high frequency due to the quiescent nature of stem cells [51]. Therefore, as discussed in the previous section, strategies are needed to enhance the efficiency of the site-specific gene integration.

##### Homology-independent targeted integration (HITI)

HITI makes use of the NHEJ pathway which is present in both dividing and non-dividing cells [49]. The technique relies on the superior activity of NHEJ in many cell types versus HDR which is active only during the S/G2 stages. HITI has been shown to use the non-error prone NHEJ repair mechanism for insertion of DNA [49]. Integration of DNA using HITI has shown to not create insertions or deletions at the target site [19, 21, 22]. However, analysis of integration efficiency needs to be further studied.

##### Microhomology mediated end joining (MMEJ)

This technique makes use of 5–25 base pair micro homologous sequences for the insertion of genes after nuclease cleavage of target DNA [50]. Further studies need to be done to demonstrate the integration efficiency of this technique. MMEJ occurs during the G1 and early S phase and therefore may be not a good technique for gene insertion in stem cells unless the efficiency can be enhanced during these cell cycle phases [50].

#### Base-editing

Correction of mutated bases using CRISPR/Cas9 fused with DNA modulators results in the translation of functional CFTR protein [52]. Cas9 helps in functioning of DNA modulators by inducing single strand R loop formation. Fusion of cytidine deaminase to Cas9 enables Cytidine to Thymidine base editing (CBE) while fusion of TadA heterodimer to Cas9 enables Adenine to Guanine base editing (ABE) [53]. The major advantage of base-editing is that it does not generate double stranded DNA breaks, thus avoiding risks of off-target insertions. The limitation of this technique is that recruitment of DNA modulators guided by the protospacer can act on bases within the target site that are not mutated or cause base changes at off-target sites [54–56]. However, unlike cytidine base editors, when using adenine base editors, no off- target mutations were detected [52, 56]. This principle was applied for base editing organoid cultures derived from CF patients and was shown to result in CFTR expression (52).

### Physical and immune barriers to gene delivery in vivo

In addition to the challenges specific to CRISPR/Cas9 delivery to airways, there are common barriers that must be overcome for achieving CRISPR/Cas9-mediated gene therapy in vivo. The first barrier to gene delivery into the airway is the mucus layer [57]. Foreign substances, including viruses, that are attached to the mucus layer are cleared by mucociliary action [58–60]. In CF patients, Ad and AAV vectors have been observed to be trapped by airway mucus [61]; the excessive buildup of mucus makes it harder for gene therapy vectors to reach the cell membrane. Clinically, patients are treated with mucolytic agents to loosen the mucus. Nacystelyn, for instance, has been shown to improve virus delivery [62]. Additionally, recombinant human DNase has also been used to reduce inhibition for both AAV and liposome-mediated gene transfer [63].

The second physical barrier is the accessibility to the Coxsackie Adenoviral Receptors (CAR) for Ad2 and Ad5 viruses. CARs are only present on the basal side, and the tight junctions between epithelial cells seal the passage from lumen. To circumvent this barrier, several chemicals that temporarily open the tight junctions have shown effects in allowing gene transfer. Ethylene Glycol Tetraacetic Acid (EGTA) is a calcium chelator that reversibly opens tight junction [64, 65]. Also, LipoPhosphatidylCholine (LPC) makes cell membranes more permissive [66, 67].

**Fig. 2:**
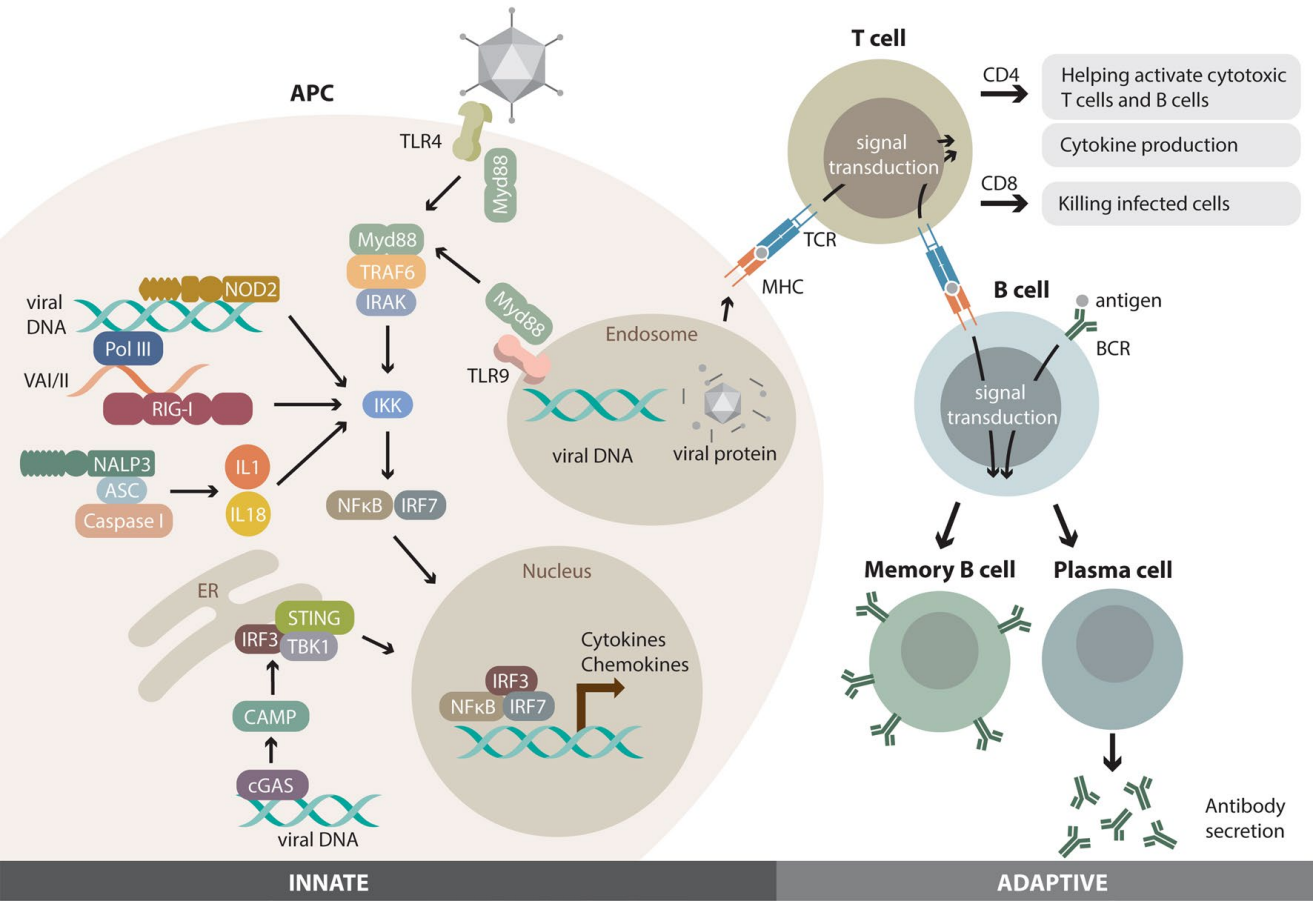
Host immune responses to adenoviral vectors. (Left side) Innate immune responses. Innate immune responses to adenoviral vectors are triggered through the recognition of adenoviral pathogen-associated molecular patterns (PAMPs), including viral capsid proteins and viral nucleic acids, by pattern recognition receptors (PRRs). PPRs involved in recognition of adenoviral PAMPS include Toll-like receptors (TLRs), such as TLR4 which interacts with viral capsid proteins at the cell surface and TLR9 which recognizes viral DNA in endosomes, and cytosolic retinoic acid-inducible gene I (RIG-I) receptor. TLR4 triggers an intracellular signaling pathway mediated by proteins including myeloid differentiation primary response gene 88 (MyD88), TNF receptor-associated factor 6 (TRAF6), IL-1 receptor-associated kinase (IRAK). This activates nuclear factor-κB (NF-κB) through a kinase IKK (I-kB kinase) and interferon (IFN) regulatory factors, such as IRF3 and IRF7, resulting in expression of cytokines and chemokines, including type I IFNs. TLR9 recognizes viral dsDNA in the endosome converging the signal to the same pathway. RIG-I recognizes viral RNA 1 and 2 (VAI/II) transcribed by RNA polymerase III and channel the information to the same pathway through IKK. In addition, another class of PRRs, nucleotide-binding oligomerization domain-like receptors (NLRs), NOD2 and NALP3, recognizes dsDNA in the cytosol and activates the inflammasome leading to the upregulation of the same pathways. Finally, the cyclic GMP-AMP synthase/ stimulator of IFN genes (cGAS/STING) pathway can also recognize cytosolic viral DNA also leading to the upregulation of the same pathway. As HD-Ad does not contain viral coding sequences, it will not produce viral RNAs. (Right side) Adaptive immune responses. T cells recognize antigen presented on major histocompatibility complex (MHC) by antigen presenting cells (APCs), commonly dendritic cells. CD4 + T cells produce cytokines and activate CD8 + T cells and B cells. CD8 + T cells kill infected cells via ADCC. Some T cells become memory T cells, which can be quickly converted into a large number of effector T cells upon re-exposure to the same antigens. B cells recognize antigen via the B cell receptor (BCR) and can be activated with or without the help of T cells. Activated B cells differentiate into plasma cells which produce antibodies, and memory B cells which have a longer life span and help mount a rapid adaptive immune response. The part of the innate immune response is adapted from “Immunology of Adenoviral Vectors in Cancer Therapy. Molecular therapy” by Shaw AR, Suzuki M, 2019, Mol Ther Methods Clin 15:418–429 [68]

Both the innate and adaptive immune responses against HD-Ad vectors are another challenge to gene delivery (Fig. 2). The innate immunity is a universal and conserved host defense mechanism [68]. Important players include airway epithelial cells and phagocytic cells like macrophages, which express Pattern Recognition Receptors (PRR), such as Toll-like Receptors (TLR), on their cell surface [69]. PRRs recognize and bind conserved non-self molecules on microbes called Pathogen-

Associated Molecular Patterns (PAMP), which activates various pathways that result in the production and release of inflammatory cytokines. One of the most studied inflammatory pathways is the Nuclear Factor-κB (NFκB) pathway. NFκB is a group of transcription factors that, when activated, promotes the transcription of pro-inflammatory cytokines, such as IL-1, IL-6, IFNγ, and TNFα [70, 71]. The elevated levels of these cytokines have been observed in both wild type and immunodeficient mice when exposed to Ad vectors, which demonstrates the important role of innate immunity [72]. In addition, pre-existing antibodies against Ad vectors are commonly found in humans although neutralization of delivered viral vectors appears not a major problem [73]. Other foreign molecules in the viral cargo also need attention, and this issue will be addressed later.

The host immune challenges can be tackled from two directions, the vector and the host (reviewed by [74]. Modifying the vector is the first direction. For example, HD-Ad was developed to mitigate host immune responses by removing all viral coding sequences. This not only reduces the inflammatory responses and improves transduction efficiency, but also increases DNA carrying capacity to 30 kb [75]. It is worth mentioning that superiority of HD-Ad vectors over the conventional Ad vectors are not fully recognized by the gene therapy community. Nevertheless, HD-Ad vectors still contain capsid proteins that can be detected by the host immune system. Another established approach is the cloak of the antigenic epitopes on viral capsid with synthetic polymers like Polyethylene Glycol (PEG) [76, 77]. In addition, since the scale of immune reaction is dose-dependent, simply lowering the vector dose can reduce inflammation.

From the host side, transient immunosuppression is a common strategy [78]. Seregin et al. 2009 have shown a dose-dependent reduction in cytokine release by adding Dexamethasone (DEX) [78]. DEX, a glucocorticoid, binds Glucocorticoid Receptors (GR) on plasma membranes; this binding triggers the translocation of GR into the nucleus where it blocks the transcription of proinflammatory genes. Cyclophosphamide, an alkylating agent, may also be used to suppress the immune responses [79]. Our group has observed significant improvement in transgene expression from readministered vectors in mouse airways via host pre-treatment with cyclophosphamide [80, 81]. Additionally, selective inhibitors of inflammatory response pathways can dampen the immune responses. For example, Caffeic Acid Phenethyl Ester (CAPE) blocks the nuclear translocation of NFκB [82]. Also, a variety of OligoDeoxyriboNucleotides (ODN) have been reported to have inhibitory activity on TLR receptors [83]. Other than immunosuppressives, non-invasive delivery methods can also minimize stress in patients. Nebulization, the delivery of therapeutic vectors in the form of inhalable mist, has been used in gene therapy clinical trials for both viral and non-viral vectors [16, 84].

## Summary

**Fig. 3:**
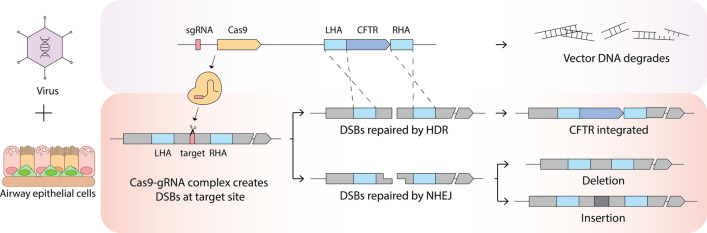
Schematic illustration of permanent correction of CFTR mutations in CF airway epithelia. (Left panel) HD-Ad vector particles carrying the Cas9/sgRNA genes and donor DNA with homology arms are delivered into airway epithelial cells, including airway basal stem cells. (Middle panel) Following the vector genome reach the nuclei of target cells, the Cas9 and its corresponding sgRNA are expressed and they work together to generate a double-stranded DNA break at the target site where the donor DNA integrates through homologous DNA strand exchanges at both left and right homology arms. If the donor DNA integration does not happen in a cell, the break is repaired through non-homologous end joining. (Right panel) The donor DNA integration compromises the vector genome integrity, leading the degradation of the rest of the vector genome and thus eliminating the undesirable expression of the Cas9 protein. While the terminally differentiated epithelial cells are eventually replaced, the permanently gene-corrected basal stem cells will be differentiated into other epithelial cells, thus perpetuating the therapeutic effects of gene correction

Recent advancements in the development of gene editing tools bring new hope for lung gene therapy. The lung is an immune-sensitive organ and it is unlikely that airway gene delivery can be repeatedly administered. CRISPR-mediated permanent gene correction of airway stem cells and progenitor cells has great potential to bring clinical benefits to patients (Fig. 3). However, major challenges discussed above have to be overcome before the real potential of the technology can be realized. Considering available strategies discussed, the challenges are not insurmountable. Our major efforts should be directed to the practically critical areas, instead of that appears to be important. Currently we think that in vivo delivery and targeting efficiency are the most important to move the field forward. We believe that HD-Ad vectors will play an important role in the clinical success of CRISPR-mediated lung gene therapy.

## Data Availability

Not applicable.

## Abbreviations

AAV: Adeno-Associated Virus
ABE: Adenine to guanine Base Editing
Ad: Adenovirus
CAPE: Caffeic Acid Phenethyl Ester
CAR: Coxsackie Adenoviral Receptor
CBE: Cytidine to thymidine Base Editing
CF: Cystic Fibrosis
CFTR: Cystic Fibrosis Transmembrane conductance Regulator
CRISPR: Clustered Regularly Interspaced Short Palindromic Repeats
DEX: Dexamethasone
DSBs: Double Stranded Breaks
EGTA: Ethylene Glycol Tetraacetic Acid
EMA: European Medicines Agency
FDA: Food and Drug Administration
GR: Glucocorticoid Receptors
HD-Ad: Helper-Dependent Adenoviral vectors
HDR: Homology Directed Repair
HITI: Homology-Independent Targeted Integration
HR: Homologous Recombination
LPC: LipoPhosphatidylCholine
MMEJ: Microhomology Mediated End Joining
NFκB: Nuclear Factor-κB
NHEJ: Non-Homologous End Joining
ODN: OligoDeoxyriboNucleotides
PAM: Protospacer Adjacent Motif
PAMP: Pathogen-Associated Molecular Patterns
PEG: Polyethylene Glycol
PRR: Pattern Recognition Receptors
sgRNA: Single guide RNA
TLR: Toll-Like Receptors
